# Comparisons of three different methods for defining sarcopenia: An aspect of cardiometabolic risk

**DOI:** 10.1038/s41598-017-06831-7

**Published:** 2017-07-26

**Authors:** Tae Nyun Kim, Man Sik Park, Eun Joo Lee, Hye Soo Chung, Hye Jin Yoo, Hyun Joo Kang, Wook Song, Sei Hyun Baik, Kyung Mook Choi

**Affiliations:** 1 Division of Endocrinology and Metabolism, Department of Internal Medicine, College of Medicine, Korea University, Seoul, Korea; 2 Department of Internal Medicine, Cardiovascular and Metabolic Disease Center, College of Medicine, Inje University, Busan, Korea; 3 Department of Statistics, College of Natural Sciences, Sungshin Women’s University, Seoul, Korea; 4 Sports Medicine, Division of Physical Education, Soonchunhyang University, A-San, Korea; 5 Health and Exercise Science Laboratory, Institute of Sports Science, Department of Physical Education, Seoul National University, Seoul, Korea

## Abstract

Appraisal of muscle mass is important when considering the serious consequences of sarcopenia in an aging society. However, the associations between sarcopenia and its clinical outcomes might vary according to the method applied in its diagnosis. We compared the relationships between cardiometabolic risk parameters and sarcopenia defined according to three different diagnostic methods using dual-energy X-ray absorptiometry (DXA) and computed tomography (CT). Appendicular skeletal muscle mass (ASM) adjusted by height squared and BMI (ASM/height^2^ and ASM/BMI) measured using DXA and thigh muscle cross-sectional area (tmCSA) adjusted by weight (tmCSA/weight) measured using CT were used as indices of muscle mass. Sarcopenia was defined as two standard deviations below either the mean ASM/height^2^, ASM/BMI, or tmCSA/weight of a young reference group. ASM/BMI and tmCSA/weight showed a negative relationship with several components of metabolic syndrome and HOMA-IR, whereas ASM/height^2^ was positively associated with theses cardiometabolic risk factors. Logistic regression analyses demonstrated that ASM/BMI-defined sarcopenia was significantly associated with increased HOMA-IR (*P* = 0.01) and prevalence of visceral obesity (*P* = 0.03) and metabolic syndrome (*P* = 0.025), while ASM/height^2^- and tmCSA/weight-defined sarcopenia were not. ASM/BMI-defined sarcopenia exhibits a closer relationship with cardiometabolic risk factors than does ASM/height^2^- or tmCSA/weight-defined sarcopenia.

## Introduction

The epidemiological trend that characterizes our generation is an aging society. Aging results in a progressive loss of muscle mass and strength, called sarcopenia, which is a Greek word meaning ‘poverty of flesh^1^’. Sarcopenia is related to physiological and metabolic deterioration, as well as functional impairment^2, 3^. Although its underlying mechanisms have not been fully clarified, several cellular and molecular mechanisms, such as inflammation, insulin resistance, vitamin D deficiency, and decreased physical activity, have been reported to play roles in the development of sarcopenia^4–6^. Interestingly, mechanisms associated with sarcopenia have been established as important risk factors for cardiometabolic diseases.

Various techniques have been used to assess muscle mass. Although bioelectrical impedance (BIA) and measurement of mid-arm muscle circumference or calf circumference may be easily applicable in the clinical field, they are often considered too inaccurate to provide reliable information^7^. In contrast, dual energy X-ray absorptiometry (DXA) determines appendicular and total body lean mass, and computed tomography (CT) estimates muscle cross sectional area (CSA).

DXA is a well-defined technique for analyzing body composition and is currently the procedure of choice for assessment of bone mineral density. Based on studies indicating that the amount of appendicular skeletal muscle mass (ASM) could be estimated using the bone-free and fat-free masses of the arms and legs as assessed with DXA^8, 9^, the most commonly used definition of sarcopenia with DXA has been proposed by Baumgartner *et al*.^10^. ASM is divided by height squared (ASM/height^2^) to adjust for body size, and sarcopenia is defined as a reduction in ASM/height^2^ relative to the values in a younger reference group. The International Working Groups on Sarcopenia (IWGS)^11^ and the European Working Group on Sarcopenia in Older People (EWGSOP)^12^ adopted this criterion based on DXA to define sarcopenia. Older men and women with low muscle mass by DXA-derived skeletal muscle mass adjusted by squared body height were shown to be three to four times more likely to report disability^10^.

However, these diagnostic criteria for sarcopenia were based on expert opinions and have not been validated with large data sets. Recently, to address this important gap, the Foundation for the National Institutes of Health (FNIH) Sarcopenia Project has developed new consensus criteria for a clinical diagnosis of sarcopenia^13^. They proposed body mass index (BMI)-adjusted appendicular skeletal muscle mass (ASM/BMI) as a muscle mass index. Hirani *et al*. reported that incident disability and mortality are associated with sarcopenia, as defined by the FNIH criteria, in elderly Australian men^14^. In our collaborative study with Australian researchers, appendicular lean mass normalized to BMI was differentially associated with metabolic syndrome, according to ethnicity^15^.

In contrast, CT and MRI are considered gold standards for clinical research, although they are expensive and difficult to adopt on a large scale^7^. A 14.7% decline in thigh muscle CSA (tmCSA), assessed by CT, was observed in older men over a period of 12 years^16^. Visser *et al*. indicated that smaller mid-thigh muscle area, evaluated by CT, was associated with poorer lower extremity performance in well-functioning older men and women^17^. Since tmCSA showed a strong association with body weight rather than with body height, tmCSA was corrected by body weight (tmCSA/weight) as a sarcopenic index of body weight burden thigh muscle mass^18^. In Japanese men, tmCSA/weight was significantly and negatively associated with carotid intima-media thickness and arterial stiffness, reliable indicators of atherosclerosis^18^. Moreover, quadriceps CSA/weight was related to postural instability, independent of age and sex^19^.

Despite the associations between sarcopenia and various important health outcomes, there has been very limited research comparing the associations between cardiometabolic risk factors and sarcopenia defined by DXA or CT. Therefore, the aim of the current study was to compare the relationships between sarcopenia indices estimated by DXA and CT (ASM/height^2^, ASM/BMI, or tmCSA/weight) and cardiometabolic risk factors known to be associated with sarcopenia, such as high sensitivity C-reactive protein (hsCRP), Homeostasis Model Assessment as an index of insulin resistance (HOMA-IR), visceral obesity, and components of metabolic syndrome, in an apparently healthy Asian population.

## Methods

### Study subjects

The current study included participants from the Korean Sarcopenic Obesity Study (KSOS), a prospective observational cohort study designed to examine the prevalence of sarcopenia and sarcopenic obesity in Korean adults with (diabetic KSOS cohort) or without diabetes (non-diabetic KSOS cohort) and to evaluate their effects on metabolic disorders and health outcomes^20, 21^. The non-diabetic cohort of adults aged ≥20 years consisted of 526 subjects (198 men and 328 women). Eligible participants in the non-diabetic KSOS cohort had no history of any type of diabetes, CVD (myocardial infarction, unstable angina, stroke, or cardiovascular revascularization), stage 2 hypertension (resting blood pressure, ≥160/100 mmHg), malignant disease, or severe renal or hepatic disease. Medical histories and lifestyle information were collected by personal interview using a detailed questionnaire^22^. All participants provided written informed consent, and the study was approved by the Korea University Institutional Review Board (IRB No.: MD0715) conforming to the Declaration of Helsinki of the World Medical Association. All methods were performed in accordance with the nationally approved guidelines and regulations.

We analyzed the data of 490 participants who had complete body compositional analysis data by a combination of both DXA and CT to define sarcopenia. Therefore, this study included 125 healthy young adults aged between 20 and 39 years (our reference group) and 365 participants aged 40 years or older (the population of interest).

### Clinical and laboratory measurements

BMI was calculated as the weight/height^2^ (kg/m^2^), and waist circumference was measured at the midpoint between the lower border of the rib cage and the iliac crest. All blood samples were obtained in the morning after a 12-hour overnight fast and were immediately stored at −80 °C for subsequent assays. Serum triglycerides and high-density lipoprotein (HDL) cholesterol levels were determined enzymatically using a chemistry analyzer (Hitachi 747; Tokyo, Japan). Low-density lipoprotein (LDL) cholesterol levels were calculated according to the Friedewald formula^23^. A glucose oxidase method was used to measure fasting plasma glucose (FPG), and an immunoradiometric assay (DIAsource Diagnostics, Nivelles, Belgium) was used to measure insulin levels. Insulin resistance was calculated using HOMA-IR^24^. hsCRP levels were measured by Latex-enhanced Turbidometric Immunoassay (HiSens hsCRP LTIA; HBI Co., Ltd.) with an interassay coefficient of variation of 7.2%. Serum 25[OH]D levels were measured using radioimmunoassay kits (DIAsource Diagnostics, Nivelles, Belgium) with quality control materials provided by the manufacturer. Metabolic syndrome was defined according to criteria established by the National Cholesterol Education Program Adult Treatment Panel III, using the adjusted waist circumference for Asians^25^. Accordingly, participants with three or more of the following five criteria were defined as having metabolic syndrome: (i) abdominal obesity based on waist circumference (defined as Asian specific waist circumference cut-off values ≥90 cm for men and ≥80 cm for women), (ii) systolic blood pressure ≥130 mmHg, diastolic blood pressure ≥85 mmHg, or use of antihypertensive medication, (iii) elevated FPG (≥5.6 mmol/L), (iv) hypertriglyceridemia (≥1.7 mmol/L), and (v) low serum HDL–cholesterol <1.03 mmol/L in men and <1.29 mmol/L in women.

### Measurement of body composition

A whole body DXA scan was performed for each patient to measure regional lean mass (kg), total body fat (kg), and total body fat percentage (%) using fan-beam technology (Hologic Discovery A, Hologic; Bedford, MA, USA). ASM (kg) was defined as the sum of the lean soft tissue mass for the arms and legs, following the method of Heymsfield *et al*.^8^. In addition, the tmCSA was quantified by CT (Brilliance 64, Philips Medical Systems, Cleveland, OH). A cross-sectional scan of the left thigh, halfway between the pubic symphisis and the inferior condyle of the femur, was performed, and the mid-thigh muscle CSA was obtained, as previously described^26^. Skeletal muscle was determined by measuring the mean value of the pixels within an attenuation range of 0 to 100 Hounsfield units to exclude most of the intermuscular, or “marbled,” adipose tissue in the analysis. However, omission of −29 to 0 HU range may fail to account for some significant proportion of the total muscle cross-sectional area in some individuals. Meanwhile, visceral fat area (VFA) was calculated from a 10-mm CT slice scan image between the fourth and fifth lumbar vertebrae, obtained during suspended respiration. VFA was quantified by delineating the intra-abdominal cavity at the internal aspect of the abdominal and oblique muscle walls surrounding the cavity and the posterior aspect of the vertebral body. Fat attenuation was determined by measuring the mean value of the pixels within a range of −190 to −30 Hounsfield units (HU).

### Definitions of sarcopenia and visceral obesity

Sarcopenia was defined using three criteria (two criteria by DXA and one criterion by CT). The first criterion was an ASM/height^2^ less than two standard deviations (SDs) below the sex-specific young adult reference value^27^. The sex-specific cut-off points of ASM/height^2^ were 7.45 kg/m^2^ in men and 5.23 kg/m^2^ in women, which were slightly different from our previous results due to the exclusion of subjects without CT data. The second criterion was an ASM/BMI less than two SDs below the gender-specific mean value of the young reference group. The ASM/BMI cut-off points for sarcopenia were 0.90 and 0.63 for men and women, respectively. Last, CT-defined sarcopenia was defined as tmCSA/weight less than two SDs below the tmCSA/weight distribution in a young reference group for both men and women^19^. The cut-off values of tmCSA/weight were 1.58 cm^2^/kg for men and 1.25 cm^2^/kg for women. On the other hand, visceral obesity was defined as a VFA ≥100 cm^2^, which is known to be highly associated with metabolic impairment^28^.

### Statistical analysis

Numerical data are expressed as means ± standard deviation or median [inter-quartile range]. Categorical variables are presented as percentage. Differences in quantitative variables between two groups were investigated using the independent two-sample *t*-test or the Mann-Whitney U-test, and Pearson’s Chi-square test was used to test for differences in the distribution of categorical variables. Cohen’s κ coefficient is used to assess the degree of agreement between different sarcopenia definitions. We specified sarcopenia as binary categories and estimate the gender-specific coefficients^29^. A value of 0 implies no agreement beyond chance, whereas a value of 1 corresponds to a perfect agreement between the two methods. Spearman’s partial correlation analysis adjusting for age and gender was performed to determine the relationships between metabolic parameters and sarcopenia indices, such as ASM/height^2^, ASM/BMI, and tmCSA/weight. Multiple linear regression analysis using each of the sarcopenia indices as a dependent variable was conducted to determine the relative contributions made by each variable to the outcome variable. Age, gender, alcohol consumption, smoking status, physical activity, systolic and diastolic blood pressure, total cholesterol, triglycerides, HDL-cholesterol, HOMA-IR, hsCRP, and 25[OH]D were used as independent variables. Statistically significant independent variables were chosen by means of a stepwise variable selection approach. Logistic regression models were used to evaluate the relationship between sarcopenia defined by DXA or CT as a dependent variable and each of the following independent variables, expressed as odds ratio (OR) with 95% confidence intervals (CI): individual metabolic syndrome components, presence of visceral obesity and metabolic syndrome, hsCRP, HOMA-IR, and 25[OH]D levels after controlling for age, gender, smoking status, alcohol consumption, and physical activity. Finally, factors related to the presence of metabolic syndrome were analyzed by multiple logistic regression analyses with a stepwise backward procedure. All statistical outcomes based on two-sided tests were obtained using SPSS for Windows (Version 12.0, SPSS Inc., Chicago, IL, USA). A *P*-value < 0.05 was regarded as significant.

## Results

**Figure 1:**
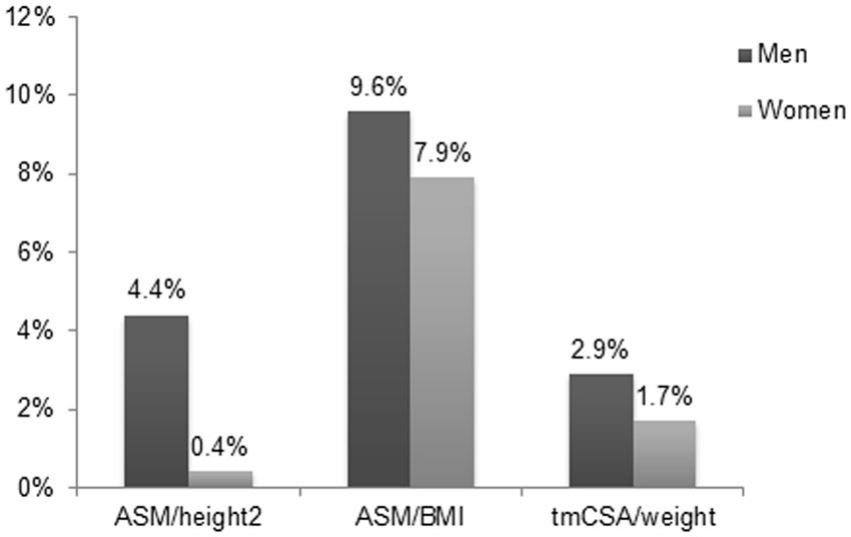
Prevalence (%) of ASM/height^2^-, ASM/BMI-, and tmCSA/weight-defined sarcopenia according to gender.

In this cohort of 365 subjects aged 40 years and older (136 men and 229 women), the average age was 58.5 ± 9.6 years, and there were no significant age differences between men and women (58.6 ± 10.7 vs 58.5 ± 9.6, *P* = 0.976). The prevalence of sarcopenia defined by ASM/height^2^ index was 1.9% and was higher among men (4.4%) than among women (0.4%). In contrast, when defined by ASM/BMI, there was no significant difference in the prevalence of sarcopenia (9.6% in men, 7.9% in women, and 8.5% across both groups). Applying tmCSA/weight, the prevalence of sarcopenia was 2.9% in men, 1.7% in women, and 2.2% in total (Fig. 1). The Cohen’s κ coefficient between the two DXA-defined criteria (ASM/height^2^ and ASM/BMI) was 0.38 in men, 0.09 in women, and 0.24 in total. By comparing the three different criteria, the degree of consensus among them demonstrated similar trends of low values in females and moderate values in males.

Table 1 shows the clinical and metabolic parameters according to the three categories of DXA- and CT-defined sarcopenia. DXA and CT, as well as height and weight adjustments, identified sarcopenic populations with different characteristics; although not statistically significant, subjects fulfilling DXA-defined and the square of height-adjusted sarcopenia criteria (ASM/height^2^) were older and had lower BMI, waist circumference, and prevalence of metabolic syndrome compared with subjects without sarcopenia. In contrast, subjects fulfilling ASM/BMI-defined or tmCSA/weight-defined sarcopenia criteria had higher BMI and lower HDL-cholesterol than those without sarcopenia. Additionally, serum 25[OH]D level tended to be lower in subjects with ASM/BMI-defined or tmCSA/weight-defined sarcopenia compared to those without sarcopenia. Defining sarcopenia based on ASM/BMI of DXA-defined sarcopenia criteria, HOMA-IR (2.3 [2.0, 3.3] vs. 1.9 [1.4, 2.6], *P* = 0.001) and serum hsCRP level (0.9 [0.3, 1.7] vs. 0.4 [0.2, 0.9], *P* = 0.001) were significantly higher in subjects with sarcopenia than in those without sarcopenia. Only subjects who fulfilled the criteria of ASM/BMI-defined sarcopenia had significantly higher prevalence of visceral obesity (*P* = 0.008) and metabolic syndrome (*P* = 0.005) relative to those without sarcopenia.

**Table 1:** Clinical characteristics of study subjects stratified by sarcopenia defined using DXA and CT.

Tool	DXA											CT				
Index of sarcopenia	ASM/height^2^						ASM/BMI					tmCSA/weight				
Clinical variables	No sarcopenia (n = 358)		Sarcopenia (n = 7)		*P*-value		No sarcopenia (n = 334)		Sarcopenia (n = 31)		*P*-value	No sarcopenia (n = 357)		Sarcopenia (n = 8)		*P*-value
Age (years)	58.4	± 9.5	63.7	± 15.3	0.151		58.1	± 9.7	63.6	± 8.2	0.001	58.4	± 9.6	64.5	± 8.2	0.074
Male gender (n (%))	130	(36.3)	6	(85.7)	0.007		122	(36.5)	13	(41.9)	0.551	131	(36.7)	4	(50.0)	0.441
Alcohol consumption (%)	196	(54.7)	5	(71.4)	0.380		175	(52.4)	26	(83.9)	0.001	194	(54.3)	7	(87.5)	0.062
Current smoker (%)	67	(18.7)	3	(42.9)	0.108		65	(19.5)	5	(16.1)	0.652	68	(19.0)	2	(25.0)	0.672
Physical activity (%)	233	(65.1)	5	(71.4)	0.727		218	(65.3)	20	(64.5)	0.933	235	(65.8)	3	(37.5)	0.096
Body mass index (kg/m^2^)	24.6	± 3.2	22.0	± 3.7	0.108		24.3	± 2.9	27.5	± 4.4	<0.001	24.5	± 3.1	27.9	± 4.0	0.003
Waist circumference (cm)	85.2	± 8.1	81.6	± 8.9	0.324		84.7	± 7.8	89.8	± 9.5	0.007	85.0	± 8.0	90.8	± 10.3	0.160
Systolic BP (mmHg)	124.4	± 12.8	127.9	± 10.4	0.419		124.4	± 12.9	125.5	± 11.3	0.624	124.5	± 12.6	124.8	± 17.3	0.966
Diastolic BP (mmHg)	80.7	± 9.9	85.1	± 10.1	0.291		80.9	± 9.9	79.6	± 9.7	0.481	80.9	± 9.9	77.1	± 10.3	0.342
Total cholesterol (mmol/L)	4.9 [4.3, 5.4]	5.1 [4.7, 5.9]	0.315		4.9 [4.3, 5.4]	5.0 [4.5, 5.3]	0.703		4.9 [4.3, 5.4]	4.7 [3.5, 5.9]	0.508					
HDL cholesterol (mmol/L)	1.4 [1.2, 1.7]	1.3 [1.2, 1.5]	0.429		1.4 [1.2, 1.7]	1.3 [1.2, 1.5]	0.029		1.4 [1.2, 1.7]	1.2 [1.1, 1.3]	0.034					
Triglycerides (mmol/L)	1.3 [0.9, 1.8]	1.2 [1.2, 1.7]	0.493		1.3 [0.9, 1.8]	1.6 [1.2, 1.9]	0.063		1.3 [0.9, 1.8]	1.7 [1.3, 1.9]	0.284					
FPG (mmol/L)	5.2 [4.9, 5.8]	5.2 [5.1, 5.7]	0.532		5.2 [4.9, 5.7]	5.3 [5.0, 6.1]	0.128		5.2 [4.9, 5.8]	5.4 [5.2, 6.2]	0.238					
HOMA-IR	1.9 [1.4, 2.6]	1.9 [1.7, 2.9]	0.547		1.9 [1.4, 2.6]	2.3 [2.0, 3.3]	0.001		1.9 [1.4, 2.6]	2.5 [1.9, 3.0]	0.098					
hsCRP (mg/L)	0.4 [0.2, 1.0]	0.9 [0.3, 2.7]	0.217		0.4 [0.2, 0.9]	0.9 [0.3, 1.7]	0.001		0.4 [0.2, 1.0]	0.8 [0.3, 1.6]	0.259					
25(OH)D (mmol/L)	28.9 [20.9, 42.2]	30.4 [24.0, 44.5]	0.858		29.9 [21.2, 43.2]	25.2 [18.0, 35.8]	0.071		30.0 [21.2, 42.6]	21.6 [17.3, 27.3]	0.098					
Presence of MetS (%)	118	(33.0)	0	(0)	0.065		101	(30.2)	17	(54.8)	0.005	115	(32.2)	3	(37.5)	0.752
Presence of visceral obesity (%)	235	(65.6)	4	(57.1)	0.639		212	(63.5)	27	(87.1)	0.008	232	(65.0)	7	(87.5)	0.185
ASM (kg)	21.3	± 4.7	16.8	± 2.8	0.005		21.4	± 4.7	18.7	± 3.4	0.002	21.2	± 4.7	20.6	± 3.0	0.603
ASM/height^2^ (kg/m^2^)	8.2	± 1.2	6.6	± 0.8	0.002		8.2	± 1.2	7.8	± 1.3	0.112	8.2	± 1.2	7.8	± 0.9	0.276
ASM/BMI (kg/kg/m^2^)	0.87	± 0.17	0.79	± 0.20	0.347		0.88	± 0.17	0.69	± 0.12	<0.001	0.87	± 0.17	0.75	± 0.11	0.023
tmCSA/weight (cm^2^/kg)	1.8	± 0.3	1.8	± 0.3	0.999		1.9	± 0.3	1.7	± 0.3	0.001	1.9	± 0.3	1.3	± 0.2	<0.001

25[OH]D, 25-hydroxyvitamin D; ASM, appendicular skeletal muscle mass; BMI, body mass index; BP, blood pressure; FPG, fasting plasma glucose; HDL, high-density lipoprotein; HOMA-IR, homeostasis model assessment of insulin resistance; hsCRP, high-sensitivity C-reactive protein; LDL, low-density lipoprotein; MetS, metabolic syndrome; tmCSA, thigh muscle cross-sectional area.

Data are presented as mean ± SD, median [inter-quartile range] or n (%), as appropriate.

*P*-values represent differences between sarcopenia and non-sarcopenia as determined by Student’s t-test or Wilcoxon rank test for continuous variables, and Fisher’s exact test or Pearson’s Chi-square test for categorical variables.

After adjusting for age and gender, ASM/height^2^ of DXA-derived muscle mass indices was positively correlated with BMI (*r* = 0.67, *P* < 0.001), whereas DXA-derived ASM/BMI and CT-derived index of muscle mass (tmCSA/weight) were negatively correlated with BMI (*r* = *−*0.42, *P* < 0.001 and *r* = *−*0.44, *P* < 0.001, respectively). Moreover, ASM/height^2^ was positively correlated with measures of adiposity, systolic and diastolic blood pressure, triglycerides, FPG, and HOMA-IR, while ASM/BMI and tmCSA/weight were negatively correlated with waist circumference, serum triglycerides level, HOMA-IR, and hsCRP and positively correlated with HDL cholesterol (Supplementary Table 1). Interestingly, ASM/BMI index was positively correlated with all of muscle indices such as ASM, tmCSA, ASM/height^2^ and tmCSA/weight, whereas tmCSA/weight index was not correlated with ASM and ASM/height^2^ among muscle indices.

Multiple regression analyses were performed using each index of muscle mass defined by DXA or CT as a dependent variable and clinical and metabolic parameters as independent variables (Table 2). ASM/height^2^ was independently and negatively associated with age (*P* < 0.001) and positively associated with systolic blood pressure (*P* < 0.001) and serum triglycerides (*P* < 0.001). In contrast, CT-derived index of muscle mass (tmCSA/weight) was independently and negatively associated with alcohol consumption (*P* = 0.001), systolic blood pressure (*P* < 0.001), and serum triglycerides (*P* = 0.010) and positively associated with 25[OH]D level (*P* = 0.023). HOMA-IR and physical activity were independently associated with both ASM/BMI and tmCSA/weight, while age was negatively associated with ASM/BMI, but not with tmCSA/weight.

**Table 2:** Multiple linear regression analysis to identify clinical and metabolic variables associated with ASM/height^2^, ASM/BMI, or tmCSA/weight.

DXA					CT	
	ASM/height^2^		ASM/BMI		tmCSA/weight	
	Coefficient (SE)	*P*	Coefficient (SE)	*P*	Coefficient (SE)	*P*
Age (years)	−0.022 (0.005)	<0.001	−0.003 (0.001)	<0.001	−0.002(0.001)	0.084
Gender (M/F)	1.424 (0.100)	<0.001	0.268 (0.013)	<0.001	0.315 (0.027)	<0.001
Physical activity			0.042 (0.011)	<0.001	0.079 (0.023)	<0.001
Alcohol consumption			−0.021 (0.013)	0.103	−0.071 (0.026)	0.007
Systolic blood pressure (mmHg)	0.015 (0.004)	<0.001	−0.001 (0.001)	0.085	−0.002 (0.001)	0.007
Total cholesterol (mmol/L)	−0.128 (0.058)	0.027				
Triglycerides (mmol/L)	0.224 (0.058)	<0.001			−0.034 (0.012)	0.005
HOMA-IR			−0.009 (0.002)	<0.001	−0.020 (0.005)	<0.001
HDL-cholesterol (mmol/L)	0.245 (0.153)	0.110				
25(OH)D (mmol/L)					0.002 (0.001)	0.023
Adjusted *R* ^*2*^	47.6%		67.4%		48.0%	

The following covariates were considered independent variables prior to stepwise variable selection approach: age, gender, alcohol consumption, smoking status, physical activity, systolic blood pressure, total cholesterol, triglycerides, HDL-cholesterol, HOMA-IR, hsCRP, 25(OH)D values.

To investigate whether DXA- or CT-defined sarcopenia was related independently to markers of inflammation and insulin resistance and the presence of visceral obesity and metabolic syndrome, multiple logistic regression analysis was performed (Table 3). After adjusting for age, gender, current smoking, alcohol consumption, and physical activity, ASM/BMI-defined sarcopenia was independently and positively associated with BMI (*P* < 0.001), HOMA-IR (*P* = 0.010), waist circumference (0.004) and presence of visceral obesity (*P* = 0.030), and was negatively associated with ASM (*P* < 0.001). Although ASM/BMI defined sarcopenia was not independently associated with each metabolic syndrome components, it was independently associated with presence of metabolic syndrome (OR 2.454, CI 1.118, 5.388, *P* = 0.025). In contrast, although BMI was independently and negatively associated with ASM/height^2^-sarcopenia and was positively associated with tmCSA/weight, ASM/height^2^-defined and tmCSA/weight-defined sarcopenia were not independently associated with presence of visceral obesity or metabolic syndrome or with HOMA-IR, hsCRP, or 25[OH]D value.

**Table 3:** Logistic analysis to evaluate relationships between sarcopenia defined by DXA or CT and clinical, metabolic, and body composition variables including presence of visceral obesity and metabolic syndrome.

	DXA-defined sarcopenia				CT-defined sarcopenia		
	ASM/height^2^		ASM/BMI			tmCSA/weight	
	OR (95% CI)^†^	*P*	OR (95% CI)^†^	*P*		OR (95% CI)^†^	*P*
BMI	0.691 (0506–0.945)	0.021	1.340 (1.181–1.520)	<0.001		1.256 (1.034–1.526)	0.022
Waist circumference	0.892 (0.798–0.997)	0.045	1.073 (1.022–1.125)	0.004		1.061 (0.975–1.154)	0.168
ASM	0.088 (0.008–0.939)	0.044	0.682 (0.574–0.810)	<0.001		0.912 (0.709–1.173)	0.472
Systolic blood pressure	1.004 (0.941–1.072)	0.902	0.991 (0.959–1.023)	0.565		0.983 (0.926–1.044)	0.575
Triglycerides	0.820 (0.302–2.225)	0.697	1.207 (0.787–1.852)	0.388		0.858 (0.307–2.395)	0.769
HDL-cholesterol	1.703 (0.143–20.206)	0.673	0.476 (0.131–1.732)	0.260		0.173 (0.009–3.176)	0.237
Fasting plasma glucose	0.735 (0.241–2.243)	0.589	1.174 (0.859–1.604)	0.313		1.248 (0.641–2.432)	0.515
HOMA-IR	0.926 (0.582–1.474)	0.747	1.226 (1.050–1.431)	0.010		1.146 (0.991–1.324)	0.066
hsCRP	1.103 (0.953–1.277)	0.189	1.036 (0.889–1.208)	0.650		1.007 (0.739–1.372)	0.964
25(OH)D	0.995 (0.938–1.056)	0.877	0.976 (0.947–1.005)	0.108		0.949 (0.879–1.024)	0.179
Presence of visceral obesity	0.357 (0.068–1.881)	0.224	3.443 (1.126–10.523)	0.030		2.431 (0.274–21.558)	0.425
Presence of Metabolic syndrome	0	0.943	2.454 (1.118–5.388)	0.025		0.981 (0.216–4.462)	0.980

CI, confidence interval; OR, odds ratio.

^†^Each of the independent variables is included in the logistic regression model after adjusting for age, gender, current smoking, alcohol consumption, and physical activity.

Because few participants with sarcopenia defined by ASM/height^2^ and tmCSA/weight may limit statistical power, we reanalyzed the relationship between each of three different sarcopenia and cardiometabolic risk factors using 1 SD below sex-specific mean for young reference group as another cutoff value of sarcopenia. Like ASM/BMI-defined sarcopenia, tmCSA/weight-defined sarcopenia was independently associated with obesity indices such as BMI and waist circumference, HOMA-IR and presence of visceral obesity especially in women (Supplementary Table 2), while the association between cardiometabolic risk factors and sarcopenia was non-significant in men when sarcopenia was defined by tmCSA/weight (Supplementary Table 3). However, contrary to our expectations, tmCSA/weight-defined sarcopenia was associated with higher ASM in women. In men, tmCSA/weight-defined sarcopenia was not significantly associated with ASM. Whereas ASM/BMI-defined sarcopenia was significantly associated with higher BMI and lower ASM in each sex using ASM/BMI-1SD of a sex-specific young reference group as a cut-off point of sarcopenia. Meanwhile, multiple logistic regression analysis showed that ASM/BMI defined sarcopenia was a risk factor for the presence of metabolic syndrome both in addition to systolic blood pressure, triglycerides, HDL-cholesterol, FPG, and ASM both in men and in women (Supplementary Table 4).

## Discussion

Sarcopenia is known to be associated with increased mortality, as well as functional decline and disability^30, 31^. Assessment of muscle mass has pivotal clinical importance in the context of increasing awareness of the far-reaching consequences of sarcopenia. Aim of the present cohort study was to compare the relation between sarcopenia as defined by three different methods and cardiometabolic risk factors. We found that sarcopenia as defined by DXA-based appendicular muscle mass adjusted for BMI (ASM/BMI) was associated with insulin resistance, visceral obesity and metabolic syndrome while sarcopenia defined by DXA based ASM adjusted for height^2^ (ASM/height^2^) and CT-defined sarcopenia adjusted for weight (tmCSA/weight) were not. Moreover, the prevalence of sarcopenia varied considerably within the same cohort when we applied different indices to define sarcopenia. We observed that the prevalence of sarcopenia was highest when defined by ASM/BMI.

Previous studies about the impact of sarcopenia on cardiometabolic risk factors have not shown consistent results. Aubertin-Leheudre *et al*. found that obese women without sarcopenia had increased cardiometabolic risk factors, such as a worse lipid profile and increased visceral fat, than did obese women with sarcopenia defined using the DXA method and adjusting for height squared^32^. Therefore, they concluded that sarcopenia appears to be associated with lower risk factors predisposing cardiovascular disease in obese postmenopausal women^32^. However, reduced muscle mass assessed using CT and adjusting for weight (tmCSA/weight) was reported to be independently associated with arterial stiffness in a general male population^18^. This finding suggests that sarcopenia diagnosed based on tmCSA using CT may lead to higher cardiovascular risk. In the present study, tmCSA/weight index was significantly correlated with metabolic syndrome related parameters including BMI even after adjusting for age and gender. In addition, when tm/CSA/weight-1SD of a sex-specific young reference group was used as a cut-off point of sarcopenia, tmCSA/weight-defined sarcopenia was significantly associated with higher ASM as well as higher BMI and HOMA-IR especially in women. These results suggest that tmCSA/weight may be more of an index of obesity rather than one of muscle in women. Although the FNIH definition with ASM/BMI is the most recent evidence-based criterion, and low ASM/BMI may have predictive value for mortality^13, 33^, little is known about the association between ASM/BMI-defined sarcopenia and metabolic derangement. In the present study, we examined an apparently healthy population without diabetes or cardiovascular disease, adjusting for body size simultaneously using height squared, BMI, or weight and using accurate methods of DXA and CT to define sarcopenia. We compared three different indices of sarcopenia using the cutoff points recommended by several major working groups on sarcopenia (two SDs below the mean levels of a young reference group) with regard to cardio-metabolic risk in this study.

Recently, there has been much debate regarding the relative strengths and weaknesses of the varying definitions of sarcopenia. Baumgartner *et al*. were the first to define sarcopenia as DXA-derived ASM/height^2^ two SD below the mean of a young reference group^10^. Although Baumgartner’s index (ASM/height^2^) has been used extensively to estimate total or regional skeletal muscle mass in adults and has been validated for this application in older subjects^34^, Newman *et al*. indicated that the ASM/height^2^ index primarily identified individuals with low BMI as sarcopenic and could have the limitation of underestimating sarcopenia in overweight or obese individuals^35^. In the present study, ASM/height^2^ of DXA-derived indices of muscle mass showed a positive relationship with metabolic syndrome components, including BMI, waist circumference, systolic and diastolic blood pressure, and triglycerides, even after adjusting for age and gender. As a result, the ASM/height^2^ index exhibits a positive association with cardiovascular risk factors, which is consistent with previous cross-sectional studies^35–37^. In contrast, our data indicated that among a set of cardiometabolic risk factors, insulin resistance, metabolic syndrome and visceral obesity appeared as independent predictors of sarcopenia as identified by ASM/BMI.

The etiology of sarcopenia is multifactorial and not fully understood. Many explanations for sarcopenia have been proposed, such as a reduction in anabolic hormone, dysregulation of cytokine secretions, a modification in the inflammatory state, insulin resistance, vitamin D deficiency, and decreased physical activity^38, 39^. Insulin resistance is associated with metabolic syndrome, which leads to type 2 diabetes and cardiovascular disease. In our previous study, type 2 diabetes was independently associated with increased risk of sarcopenia^21^. HOMA-IR, as a marker for insulin resistance, was independently and negatively associated with both muscle mass indices (ASM/BMI and tmCSA/weight) after correcting for potential confounding factors. However, HOMA-IR was independently implicated only with ASM/BMI-defined sarcopenia rather than with tmCSA/weight-defined sarcopenia in Korean adults. In addition, we observed an association between ASM/BMI-defined sarcopenia and the presence of metabolic syndrome, whereas ASM/height^2^- and tmCSA/weight-defined sarcopenia did not show such an independent relationship. Visceral obesity induces dysregulation of inflammatory cytokines, such as TNF-α and interleukin-6 (IL-6), which have catabolic effects on skeletal muscle^40^. We reported that visceral obesity was related to future loss of skeletal muscle mass in a longitudinal study^41^.

Physical activity is a pivotal factor associated with both sarcopenia and cardiometabolic indices. Muscle mass, which is increased by a high degree of physical activity, may be a proxy for lifestyle variables. This study found that physical activity is independently and positively associated with ASM/BMI and tmCSA/weight of muscle mass indices as a continuous variable although physical activity was not independently associated with the presence of sarcopenia as a category. In contrast, many previous studies have reported that muscle mass itself is significantly associated with disability and chronic metabolic disorders, even after adjusting for confounding variables, including lifestyle factors^10, 21^. The present study also demonstrated that different associations with risk factors according to the method of definition persisted after adjusting for lifestyle variables, including physical activity.

There are several limitations to this study. First, we used baseline data from an ongoing prospective cohort study, which did not allow us to determine causality. Analysis of follow-up data might provide appropriate information about causal relationships. Sarcopenia is associated with physical inactivity and exacerbate obesity-associated insulin resistance, and therefore could promote the development of metabolic syndrome^12, 40, 42^. We found that sarcopenia and metabolic syndrome are deeply interwined and could cause adverse effects on each other. Second, our cohort was composed of relatively well-functioning Asian elderly adults, which limits the ability to generalize our study results to other ethnic groups or populations with different characteristics. Third, few participants with sarcopenia defined tmCSA/weight and ASM/height^2^ can hinder the ability to detect associations between sarcopenia and cardiovascular risk factors. In addition, statistical analyses were not performed separately for women and men in this study. Therefore, for trending, we performed multiple logistic regression analysis on the association of each of three different indices-defined sarcopenia with cardiometabolic risk factors separately in women and men using 1 SD below sex-specific mean for young reference group. Ideally, our results should be validated in study populations with larger numbers of participants with sarcopenia. Lastly, potentially relevant muscle quality measurements, such as muscle strength and gait speed, were not performed as part of this study. However, a strength of this study is that that we assessed muscle mass using the most precise techniques for detecting sarcopenia, DXA and CT, and simultaneously adjusted for body size in different ways. Furthermore, we also evaluated the potential risk factors for sarcopenia and cardiometabolic diseases, such as components of metabolic syndrome, HOMA-IR, hsCRP, vitamin D level, and presence of visceral obesity assessed by abdominal CT. Although the determination of which operational methods are most appropriate for determining the degree of low muscle mass that actually contributes to clinical outcomes for sarcopenia, including weakness and slowness, in given individuals remains inconclusive, our study revealed that ASM/BMI, the FNIH recommended muscle mass index, is more capable of showing the effects of age and cardiovascular risk factors on the presence of sarcopenia.

In conclusion, the present study demonstrates that sarcopenia diagnosed using the various muscle mass indices of ASM/height^2^, ASM/BMI, and tmCSA/weight show considerably different relationships with cardiometabolic risk factors known to be associated with the pathogenesis of sarcopenia. Cardiovascular disease risk factors, including insulin resistance index, visceral obesity, and metabolic syndrome, were closely related to ASM/BMI-defined sarcopenia rather than to ASM/height^2^- or tmCSA/weight-defined sarcopenia.

## Electronic supplementary material

Supplementary Information
