# Supplementary material for: Comparisons of three different methods for defining sarcopenia: An aspect of cardiometabolic risk
Source: Sci Rep. 2017 Jul 26;7:6491. doi: 10.1038/s41598-017-06831-7 (PMC5529503; doi:10.1038/s41598-017-06831-7)
Supplement: Supplementary file 1 — Supplementary Information [file 41598_2017_6831_MOESM1_ESM.doc]

**Comparisons of three different methods for defining sarcopenia: An aspect of cardiometabolic risk**

Tae Nyun Kim^1,2^, Man Sik Park^3^, Eun Joo Lee^2^, Hye Soo Chung^1^, Hye Jin Yoo^1^, Hyun Joo Kang^4^, Wook Song^5^, Sei Hyun Baik^1^, Kyung Mook Choi^1^

^1^Division of Endocrinology and Metabolism, Department of Internal Medicine, College of Medicine, Korea University, Seoul, Korea; ^2^Department of Internal Medicine, Cardiovascular and Metabolic Disease Center, College of Medicine, Inje University, Busan, Korea; ^3^Department of Statistics, College of Natural Sciences, Sungshin Women’s University, Seoul, Korea; ^4^Sports Medicine, Division of Physical Education, Soonchunhyang University, A-San, Korea; ^5^Health and Exercise Science Laboratory, Institute of Sports Science, Department of Physical Education, Seoul National University, Seoul, Korea

|  | DXA | | | | |  | CT | |
| --- | --- | --- | --- | --- | --- | --- | --- | --- |
|  | ASM/height^2^ adjusted for  age and gender | |  | ASM/BMI adjusted for  age and gender | |  | tmCSA/weight adjusted for age and gender | |
|  | *r* | *P* |  | *r* | *P* |  | *r* | *P* |
| BMI | 0.670 | <0.001 |  | -0.420 | <0.001 |  | -0.440 | <0.001 |
| Waist circumference | 0.479 | <0.001 |  | -0.273 | <0.001 |  | -0.410 | <0.001 |
| Systolic blood pressure | 0.206 | <0.001 |  | -0.079 | 0.135 |  | -0.129 | 0.014 |
| Diastolic blood pressure | 0.149 | 0.004 |  | 0.015 | 0.774 |  | -0.107 | 0.041 |
| Total cholesterol | -0.020 | 0.699 |  | -0.063 | 0.229 |  | -0.088 | 0.095 |
| HDL cholesterol | -0.048 | 0.357 |  | 0.111 | 0.035 |  | 0.175 | <0.001 |
| Triglycerides | 0.178 | <0.001 |  | -0.174 | <0.001 |  | -0.265 | <0.001 |
| Fasting plasma glucose | 0.148 | 0.005 |  | -0.018 | 0.727 |  | -0.080 | 0.128 |
| HOMA-IR | 0.197 | <0.001 |  | -0.238 | <0.001 |  | -0.327 | <0.001 |
| hsCRP | 0.139 | 0.008 |  | -0.188 | <0.001 |  | -0.200 | <0.001 |
| 25[OH]D | 0.041 | 0.442 |  | 0.026 | 0.618 |  | 0.126 | 0.016 |
| ASM | 0.868 | <0.001 |  | 0.458 | <0.001 |  | -0.059 | 0.261 |
| tmCSA | 0.672 | <0.001 |  | 0.215 | <0.001 |  | 0.388 | <0.001 |
| ASM/BMI | 0.194 | <0.001 |  | - | - |  | 0.385 | <0.001 |
| tmCSA/weight | 0.005 | 0.924 |  | 0.385 | <0.001 |  | - | - |

**Supplementary Table 1** Correlation analysis between DXA- or CT-derived indices of sarcopenia and clinical and metabolic variables

25[OH]D, 25-hydroxyvitamin D; ASM, appendicular skeletal muscle mass; BMI, body mass index; hsCRP, high-sensitivity C-reactive protein; LDL, low-density lipoprotein; HDL, high-density lipoprotein; HOMA-IR, homeostasis model assessment of insulin resistance; MetS, metabolic syndrome; tmCSA, thigh muscle cross-sectional area

Correlation coefficients (*r*) and *P* values were calculated using the Spearman partial correlation analysis.

**Supplementary TABLE 2** Logistic analysis to evaluate relationships between sarcopenia defined by 1 standard deviation of young reference group and cardiometabolic risk factors in women

|  | DXA-defined sarcopenia | | | | |  | CT-defined sarcopenia | |
| --- | --- | --- | --- | --- | --- | --- | --- | --- |
|  | ASM/height^2^ (n = 11, 4.8%) | |  | ASM/BMI (n = 95, 41.5%) | |  | tmCSA/weight (n = 34, 14.8%) | |
|  | OR (95% CI)† | *P* |  | OR (95% CI)† | *P* |  | OR (95% CI)† | *P* |
| BMI | 0.543 (0.398 – 0.741) | <0.001 |  | 1.276 (1.151 – 1.415) | <0.001 |  | 1.280 (1.139 – 1.438) | <0.001 |
| Waist circumference | 0.902 (0.821 – 0.992) | 0.033 |  | 1.074 (1.031 – 1.119) | 0.001 |  | 1.156 (1.090 – 1.227) | <0.001 |
| ASM | 0.369 (0.213 – 0.640) | <0.001 |  | 0.666 (0.569 – 0.778) | <0.001 |  | 1.236 (1.055 – 1.449) | 0.009 |
| Systolic blood pressure | 0.977 (0.926 – 1.030) | 0.388 |  | 1.009 (0.985 – 1.032) | 0.473 |  | 1.031 (0.999 – 1.063) | 0.057 |
| Triglycerides | 0.268 (0.061 – 1.172) | 0.080 |  | 0.969 (0.646 – 1.454) | 0.881 |  | 1.139 (0.683 – 1.900) | 0.619 |
| HDL-cholesterol | 0.802 (0.138 – 4.662) | 0.805 |  | 0.900 (0.401 – 2.017) | 0.798 |  | 0.349 (0.101 - 1.200) | 0.095 |
| Fasting plasma glucose | 0.638 (0.229 – 1.773) | 0.389 |  | 1.065 (0.757 – 1.498) | 0.720 |  | 1.424 (0.963 – 2.105) | 0.077 |
| HOMA-IR | 0.465 (0.189 – 1.144) | 0.095 |  | 1.329 (1.048 – 1.685) | 0.019 |  | 1.613 (1.218 – 2.136) | 0.001 |
| hsCRP | 1.047 (0.751 – 1.460) | 0.785 |  | 1.120 (0.969 – 1.295) | 0.124 |  | 0.946 (0.738 – 1.212) | 0.660 |
| 25(OH)D | 0.977 (0.934 – 1.023) | 0.326 |  | 0.996 (0.979 – 1.013) | 0.646 |  | 0.970 (0.942 – 0.998) | 0.039 |
| Presence of visceral obesity | 0.240 (0.057 – 1.009) | 0.051 |  | 1.907 (1.042 – 3.491) | 0.036 |  | 2.606 (1.077 – 6.306) | 0.034 |

ASM, appendicular skeletal muscle mass; BMI, body mass index; CI, confidence interval; hsCRP, highly-sensitive C reactive peptide; OR, odds ratio.

†Each of the independent variables is included in the logistic regression model after adjusting for age, gender, current smoking, alcohol consumption, and physical activity.

**Supplementary TABLE 3** Logistic analysis to evaluate relationships between sarcopenia defined as 1 SD below sex-specific mean for young reference group and cardiometabolic risk factors in men

|  | DXA-defined sarcopenia | | | | |  | CT-defined sarcopenia | |
| --- | --- | --- | --- | --- | --- | --- | --- | --- |
|  | ASM/height^2^ (n = 27, 19.9%) | |  | ASM/BMI (n = 61, 44.9%) | |  | tmCSA/weight (n = 8, 5.9% ) | |
|  | OR (95% CI)† | *P* |  | OR (95% CI)† | *P* |  | OR (95% CI)† | *P* |
| BMI | 0.443 (0.311 – 0.632) | <.001 |  | 1.155 (1.001 – 1.334) | 0.049 |  | 1.305 (0.962 – 1.770) | 0.087 |
| Waist circumference | 0.874 (0.806 – 0.947) | 0.001 |  | 1.017 (0.966 – 1.071) | 0.516 |  | 1.068 (0.963 – 1.183) | 0.212 |
| ASM | 0.294 (0.172 – 0.503) | <.001 |  | 0.599 (0.489 – 0.735) | <0.001 |  | 1.046 (0.838 – 1.306) | 0.692 |
| Systolic blood pressure | 0.975 (0.936 – 1.015) | 0.219 |  | 1.018 (0.985 – 1.052) | 0.296 |  | 1.012 (0.948 – 1.080) | 0.718 |
| Triglycerides | 0.832 (0.505 – 1.371) | 0.470 |  | 0.967 (0.701 – 1.334) | 0.838 |  | 2.035 (1.131 – 3.661) | 0.018 |
| HDL-cholesterol | 2.393 (0.600 - 9.539) | 0.216 |  | 0.606 (0.190 - 1.930) | 0.397 |  | 0.285 (0.017 - 4.874) | 0.386 |
| Fasting plasma glucose | 1.064 (0.775 - 1.462) | 0.701 |  | 0.932 (0.703 - 1.236) | 0.624 |  | 1.273 (0.809 - 2.001) | 0.297 |
| HOMA-IR | 1.050 (0.885 – 1.246) | 0.577 |  | 1.260 (0.957 – 1.659) | 0.100 |  | 1.162 (0.955 – 1.414) | 0.134 |
| hsCRP | 0.984 (0.840 – 1.152) | 0.841 |  | 0.931 (0.789 – 1.098) | 0.394 |  | 0.882 (0.535 – 1.455) | 0.624 |
| 25(OH)D | 0.995 (0.938 – 1.056) | 0.811 |  | 0.980 (0.956 – 1.005) | 0.121 |  | 0.914 (0.836 – 1.000) | 0.049 |
| Presence of visceral obesity | 0.288 (0.104 – 0.798) | 0.017 |  | 2.607 (0.986 - 6.888) | 0.053 |  | 2.008 (0.213 – 18.894) | 0.542 |

ASM, appendicular skeletal muscle mass; BMI, body mass index; CI, confidence interval; hsCRP, highly-sensitive C reactive peptide; OR, odds ratio.

†Each of the independent variables is included in the logistic regression model after adjusting for age, gender, current smoking, alcohol consumption, and physical activity.

**Supplementary Table 4** Multiple logistic regression analysis to identify clinical, metabolic, and body composition variables associated with presence of metabolic syndrome as the dependent variable

| Dependent variable:  Metabolic syndrome | Men | | |  | Women | | |  | All | | |
| --- | --- | --- | --- | --- | --- | --- | --- | --- | --- | --- | --- |
|  | Odds ratio (95% CI) | | *P*-value |  | Odds ratio (95% CI) | | *P*-value |  | Odds ratio (95% CI) | | *P*-value |
| Gender |  |  |  |  |  |  |  |  | 0.037 | (0.011-0.127) | <.001 |
| Systolic blood pressure | 1.117 | (1.052-1.185) | <.001 |  | 1.095 | (1.054-1.137) | <.001 |  | 1.097 | (1.064-1.131) | <.001 |
| Triglycerides | 2.139 | (1.201-3.811) | 0.010 |  | 7.771 | (3.203-18.854) | <.001 |  | 3.387 | (1.998-5.740) | <.001 |
| HDL cholesterol | 0.038 | (0.004-0.363) | 0.004 |  | 0.084 | (0.017-0.415) | 0.002 |  | 0.049 | (0.013-0.185) | <.001 |
| Fasting plasma glucose | 2.742 | (1.466-5.126) | 0.002 |  | 2.521 | (1.420-4.476) | 0.002 |  | 2.538 | (1.687-3.818) | <.001 |
| ASM | 1.352 | (1.136-1.609) | 0.001 |  | 1.186 | (0.984-1.430) | 0.074 |  | 1.261 | (1.119-1.422) | <.001 |
| Presnece of sarcopenia | 19.658 | (2.751-140.489) | 0.003 |  | 3.942 | (1.017-15.281) | 0.047 |  | 6.531 | (2.169-19.672) | 0.001 |

The following independent variables were considered in the multiple logistic regression model prior to the stepwise variable selection approach: age, gender, body mass index (BMI), alcohol consumption, smoking status, physical activity, systolic blood pressure, total cholesterol, triglycerides, HDL-cholesterol, HOMA-IR, hsCRP, 25(OH)D values, appendicular skeletal muscle (ASM), and presence of sarcopenia defined by ASM/BMI.
